# Genomic Characterization of *Pseudomonas syringae* pv. *syringae* Populations Affecting Sweet Cherry Orchards in Chile Reveals Local Adaptation and Virulence Signatures

**DOI:** 10.3390/plants15040552

**Published:** 2026-02-10

**Authors:** Francisco Correa, Paz Millas, Rubén Almada, Franco Figueroa, Juan Pablo Martinez, Boris Sagredo

**Affiliations:** 1 Instituto de Investigaciones Agropecuarias (INIA) Rayentué, Rengo 2940000, Chile; fcorreas23@gmail.com (F.C.); franco.figueroa.grenett@gmail.com (F.F.); jpmartinez@inia.cl (J.P.M.); 2 Instituto de Investigaciones Agropecuarias (INIA) Quilamapu, Chillan 3800062, Chile; pmillas@inia.cl; 3 Centro de Estudios Avanzados en Fruticultura (CEAF), Rengo 2940000, Chile; ralmada@ceaf.cl

**Keywords:** bacterial canker, *Prunus avium* disease, *Pseudomonas syringae* genomes, *Pseudomonas syringae* pv. *syringae*

## Abstract

*Pseudomonas syringae* pv. *syringae* (*Pss*) is an economically significant bacterial pathogen that causes canker in sweet cherry trees. In Chile, sweet cherry (*Prunus avium* L.) is a key crop whose exponential production growth has increased phytosanitary pressure. However, the genetic diversity and adaptive mechanisms of local *Pss* populations have remained poorly understood. This study characterized 41 *Pss* isolates from major Chilean production regions. Their genomes were sequenced and compared with 152 public genomes from the PG2 phylogenetic group. The analysis revealed a predominance of the PG2d subgroup among the Chilean isolates, with a population structure defined by at least 18 genomic clusters, some of which are exclusive to Chile. A characteristic feature of this entire PG2d subgroup is the presence of indole-3-acetic acid (IAA) synthesis genes (*iaaM* and *iaaH*). Furthermore, this subgroup displayed a marked increase in ancestral gene gain and loss events, indicating extensive remodeling of the shell genome and supporting a model of lineage-specific adaptive evolution. We also identified lineage-specific orthogroups, structural variants of the T-PAI pathogenicity island, and a differential distribution of Hop-type effector proteins. Furthermore, an extended copper resistance operon (*cop* and *cus* systems) was detected in a subset of strains, and a dominant lineage was found to have a dual i1-type of T6SS system. These findings highlight the local diversification of *Pss* in Chile, likely driven by agro-environmental pressures. This study provides crucial insights into the evolution, adaptation, and pathogenic potential of this important pathogen in a crop of high strategic value.

## 1. Introduction

Sweet cherry (*Prunus avium* L.) has become one of the most relevant fruit crops in Chilean agriculture, with sustained growth in cultivated area and exports over the past decade [1]. Chile currently stands as the world’s leading exporter of fresh cherries [2], making this fruit a cornerstone of the agricultural sector, especially in the central and southern regions of the country [1]. However, production intensification has increased phytosanitary pressure, with bacterial canker being one of the main diseases affecting orchard health and productivity.

Bacterial canker of sweet cherry is caused by various lineages within the *Pseudomonas syringae* complex [3], a highly diverse taxonomic group that infects a wide variety of host plants. This complex comprises numerous pathovars and has been classified into thirteen phylogenetic groups (PGs) based on multilocus and genomic analyses [4,5]. Virulent strains on sweet cherry trees have been reported in PG1 (*P. avellanae*), PG2 (*P. syringae* pv. *syringae*), PG3 (*P. amygdali* pv. *morsprunorum*), and PG7 (*P. viridiflava*), evidencing evolutionary convergence of different lineages toward infection of *Prunus* species [6]. Within this complex, the main pathogen affecting sweet cherry trees in Chile is *P. syringae* pv. *syringae* (*Pss*), which has been consistently classified within the PG2 phylogenetic group [7].

*Pss* is a pathogen with a broad host range and is one of the most aggressive members of the complex, capable of causing necrosis in buds, branches, leaves, and fruits, leading to significant economic losses in *Prunus* species [8]. Its virulence is attributed to a combination of factors, including the production of phytotoxins [9], a diverse repertoire of type III virulence effectors [3], ice nucleation ability under low temperature conditions [10], and persistence in epiphytic environments [11]. These features not only facilitate initial infection but also hinder effective field control and management.

Previous studies have shown that *P. syringae* can form structured local populations, influenced by ecological and agroclimatic factors as well as region-specific agricultural practices [6]. However, in Chile, genomic characterization of *Pss* isolates associated with cherry trees has been limited, impeding a deeper understanding of their diversity, population dynamics, and adaptive potential. This information is essential for developing effective strategies for integrated management, epidemiological monitoring, and biological control. In this context, comparative genomic analyses enable in-depth evaluation of *Pss* genetic variability, identification of virulence and resistance factors, and insight into the evolution of pathogenic lineages at both local and global scales. Moreover, studying molecular mechanisms like secretion systems, type III effector repertoires (T3SEs), and copper resistance operons may uncover how pathogens adapt to intensive agricultural conditions in Chile, where copper-based bactericides are widely used.

This study aims to genomically characterize a representative collection of *Pss* isolates from sweet cherry orchards in Chile, to phylogenetically contextualize them alongside international reference genomes, and to analyze the distribution of genes associated with virulence, adaptation, and copper resistance. Our findings contribute to a better understanding of the structural and functional diversity of this important phytopathogenic bacterium in a crop of strategic importance to Chilean agriculture.

## 2. Results

### 2.1. Genomic Classification and Population Structure of Chilean Isolates

**Figure 1:**
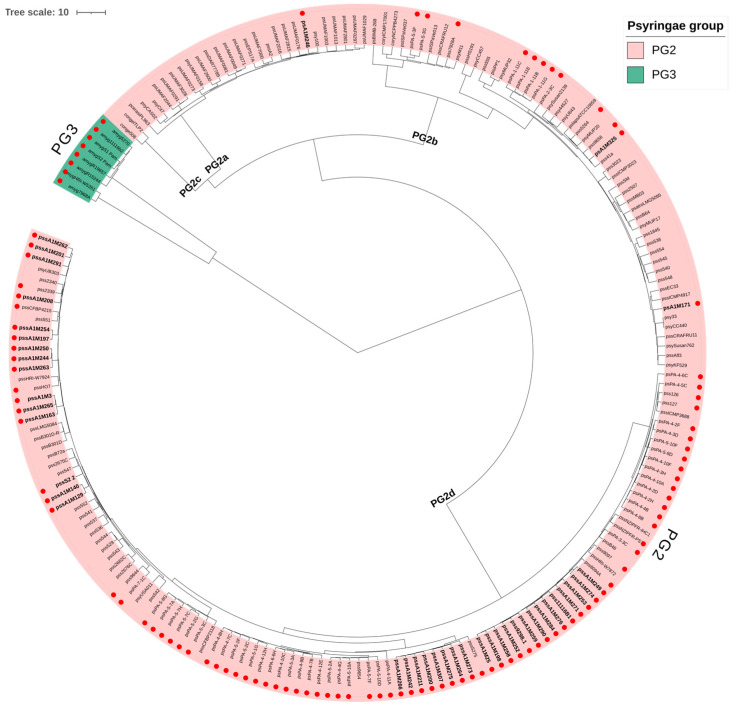
Genomic structure and diversity of *Pseudomonas syringae* pv. *syringae* (PG2d) isolates from Chilean cherry orchards. Dendrogram generated from an Average Nucleotide Identity (ANI) matrix showing the genomic relationships among *P. syringae* strains belonging to phylogroups PG2 and PG3. The dendrogram clusters most Chilean isolates within PG2d (pink) and distinguishes them from PG3 reference genomes (green). Strains isolated from *Prunus avium* are indicated with red circles. The Chilean isolates analyzed in this figure are detailed in Table 1.

A total of 201 *P. syringae* genomes were analyzed, including 41 isolates obtained from symptomatic tissues of sweet cherry (*Pr. avium*) in Chile (Table 1) and 152 reference genomes obtained from various plant hosts and geographic origins, all belonging to phylogenetic group PG2 (Table S1). Additionally, eight PG3 strains isolated from sweet cherry trees were included as outgroups for comparative analysis. Among the reference genomes, 33 correspond to complete genomes, which were used as references for comparative analyses, while the remaining are draft genomes. Although draft assemblies are less complete, they still provide valuable insights into the genomic diversity of the population. ANI analysis confirmed that Chilean *Pss* isolates grouped within PG2, with pairwise identity values ranging from 97% to 99% when compared to well-characterized reference genomes from this group (Figure 1).

Beyond confirming the assignment of Chilean *Pss* isolates to PG2, this group could be subdivided into at least four distinct phylogenetic subgroups (ANI > 96%), PG2a, PG2b, PG2c, and PG2d, as reported by Berge et al. [4]. In PG2a (*n* = 26), with only one Chilean isolate, the most representative host was *Mangifera indica* L., although strains isolated from *Pr. avium*, *Prunus cerasus* L., and legumes, such as *Phaseolus lunatus* L., were also identified. PG2b (*n* = 52) was the most host-diverse subgroup, including economically important species such as *Citrus x limon* (L.) Osbeck, *Malus domestica* Borkh, *Phaseolus vulgaris* L., *Triticum aestivum* L., and *Pr. avium*. The genus *Phaseolus* was the most frequently represented, followed by *Malus* and *Prunus*; two Chilean strains were found in this subgroup. PG2c, represented by only two strains, included hosts such as *M. domestica* and *Solanum tuberosum* (L.), with no Chilean isolates. In contrast, PG2d (*n* = 113) included the majority of Chilean *Pss* isolates (*n* = 38) and showed a strong association with *Prunus* species, particularly *Pr. avium*, followed by *Pyrus communis* L. and *Ph. vulgaris*. Notably, PG2d also included nine complete reference genomes, five of which were isolated from different *Prunus* hosts. This pattern suggests that PG2d may be adapted to woody hosts in temperate regions, especially cultivated sweet cherry trees, reinforcing its role as a dominant lineage in Chilean fruit orchards.

**Figure 2:**
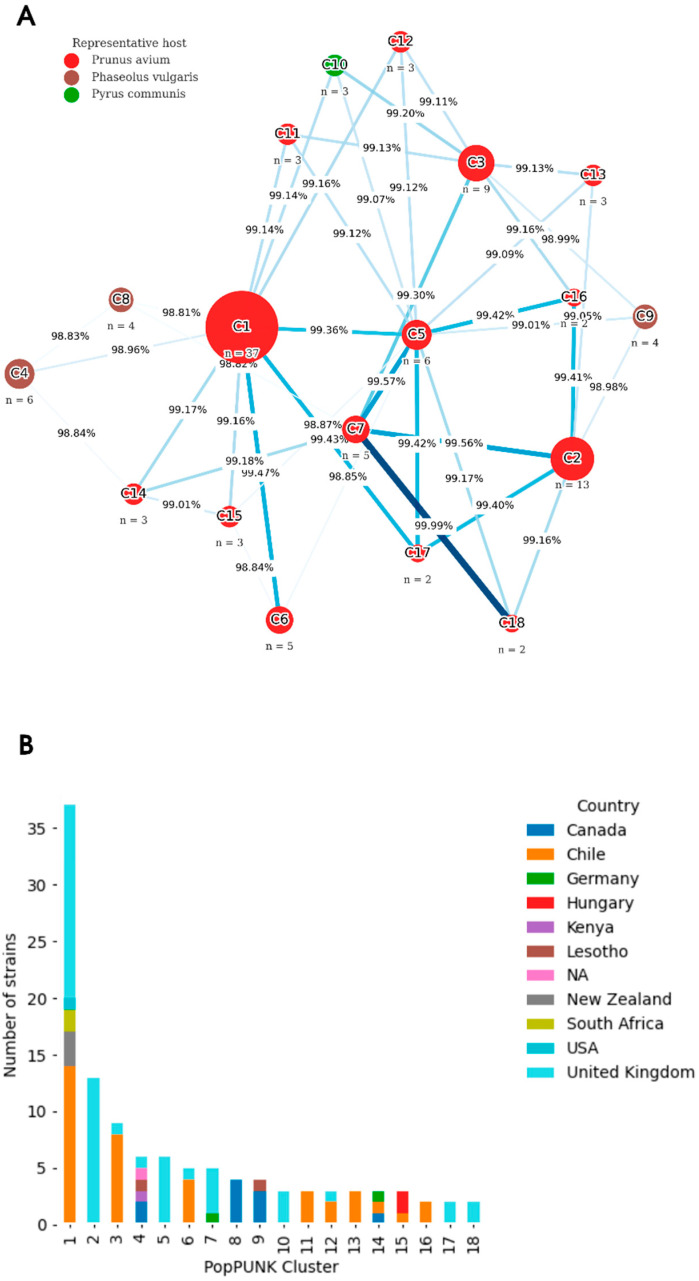
Phylogenetic clustering and geographical distribution of PG2d genomes defined by PopPUNK. (**A**) Cluster network constructed exclusively from PG2d genomes, representing inter-cluster relationships based on mean ANI values. Each node corresponds to a PopPUNK cluster, and its size is proportional to the number of genomes it contains. Edge width and color intensity indicate the mean ANI among the three closest clusters. Node colors represent the predominant host associated with each cluster: red = *Prunus avium* L., brown = *Phaseolus vulgaris* L., and green = *Pyrus communis* L. (**B**) Distribution of PG2d PopPUNK clusters by country of origin. Bars indicate the number of genomes per cluster, grouped by country. Most Chilean isolates are concentrated in clusters C1 and C3, while some clusters consist exclusively of Chilean strains.

Based on the structure genetic analysis performed by PopPUNK, which applies a Gaussian mixture model to pairwise core and accessory genome distances to identify within- and between-lineage thresholds [12], a total of 18 clusters were identified as connected components within-lineage PG2d (Figure 2A; Table 2).

Cluster 1 was the largest and most geographically diverse, grouping strains from Chile (*n* = 14), the United Kingdom, South Africa, New Zealand, and the United States (Figure 2B). Most of these isolates were associated with *Pr. avium*, although other *Prunus* species were also represented, suggesting that this cluster corresponds to a globally distributed dominant PG2d lineage. Importantly, Cluster 1 also included two complete reference genomes: *Pss*9097, isolated from *Pr. avium*, and *Pss*B48, isolated from *Prunus persica* (L.) Batsch. Cluster 2 was composed exclusively of strains from the United Kingdom, all isolated from *Pr. avium*, suggesting a localized lineage potentially adapted to specific agroclimatic conditions or cultivars in that region. In contrast, Cluster 3 consisted mainly of Chilean isolates (*n* = 8) from *Pr. avium*, along with a single strain from the United Kingdom. This composition suggests recent regional expansion or local adaptation of this lineage in Chilean orchards. Cluster 4 grouped strains isolated from *Ph. vulgaris* and other legumes, originating from Canada, Kenya, and Lesotho, suggesting a legume-specialized lineage with global distribution. This cluster also included two complete reference genomes: *Psy*USA011, isolated from stream water, and *Pss*B72a, isolated from *Ph. vulgaris.* Cluster 5 included only strains from the United Kingdom associated with *Pr. avium*, with no Chilean representation, but it was relevant as a comparative lineage within PG2d. Cluster 6 included Chilean (*n* = 4) and UK strains associated with *Pr. avium* and *Pr. cerasus*, suggesting a lineage with interregional distribution. Clusters 7 and 8 were composed exclusively of northern hemisphere isolates (UK, Canada, and Germany), associated with similar hosts such as *Prunus* and *Phaseolus*, and they had no Chilean strains. Cluster 7 contained the complete genome *Pss*CFBP2118, isolated from *Pr. cerasus*. Likewise, Clusters 9 and 10, also without Chilean isolates, are relevant reference groups. Cluster 9 grouped four *Ph. vulgaris* strains from Canada and Lesotho, indicating a legume-adapted lineage. Cluster 10 included three closely related UK strains isolated from *Pyr. Communis*; among them, the complete genome *Pss*B301D, representing a conserved lineage adapted to pear trees. Notably, Clusters 11, 13, and 16 consisted exclusively of Chilean strains, with 3, 3, and 2 isolates, respectively, all from *Pr. avium*, supporting the hypothesis of local diversification. Clusters 12, 14, and 15, with 2, 1, and 1 Chilean isolates each, respectively, also include foreign isolates, and among them, two reference genomes: *Psy*UB303, isolated from lake water (Cluster 12), and *Pss*CFBP4215, isolated from *Pr. avium* (Cluster 14). Finally, Clusters 17 and 18 were composed exclusively of UK strains, all isolated from *Pr. avium*. Importantly, Cluster 17 also contained the complete genome *Pss*9644, isolated from *Pr. avium*. These groupings represent host-specific and potentially conserved lineages, serving as comparative references to the Chilean lineages.

### 2.2. Type III and Type VI Secretion Systems Are Diverse

**Figure 3:**
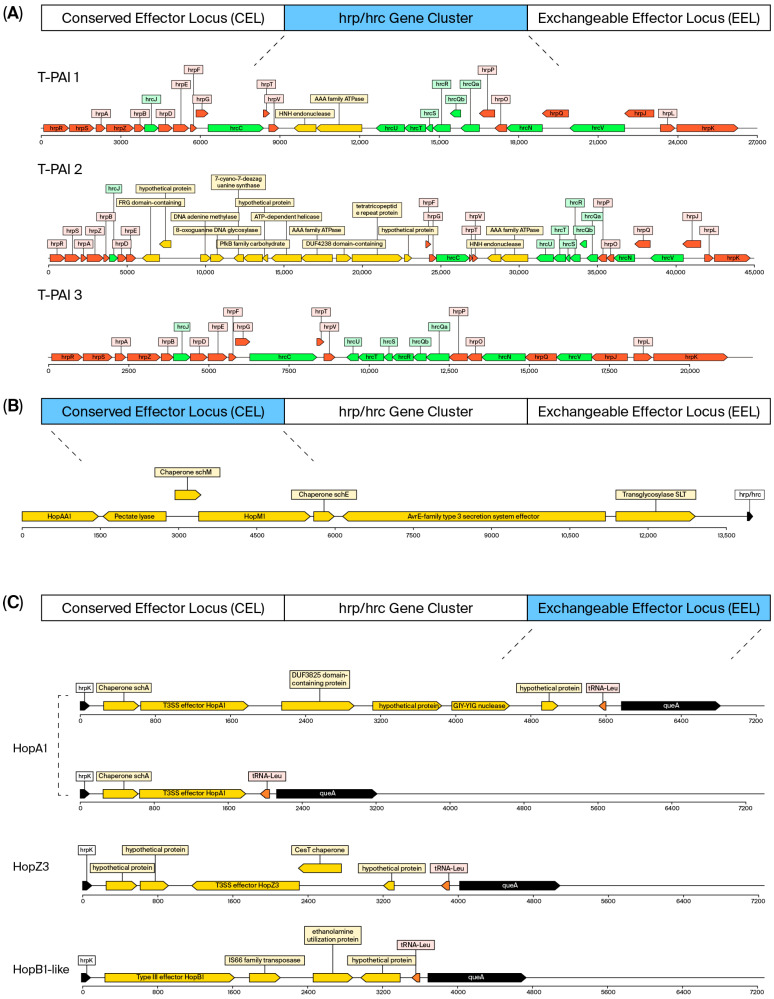
Structural organization of the type III secretion system (T3SS) in Chilean and reference PG2d isolates. (**A**) Comparative architecture of the T-PAI pathogenicity island showing three genotypes (T-PAI-1, -2, -3). T-PAI-1 is the predominant configuration, while T-PAI-2—with a 21.7 kb insertion containing 12 ORFs—is exclusive to Chilean isolates (Cluster 13). (**B**) Conserved Effector Locus (CEL) organization, including *avrE1*, *hopM1*, *hopAA1*, and their chaperones (*shcE*, *shcM*). (**C**) Exchangeable Effector Locus (EEL) variants displaying *hopA1*, *hopB-like,* and *hopZ3* effectors. Color boxes represent effector, regulatory, and hypothetical genes. The structural variability in the EEL contrasts with the conserved CEL, underscoring evolutionary diversification of the T3SS effector repertoire among *Pss* lineages.

There were both Chilean isolates and international reference strains. All isolates of *Pss* harbored genes corresponding to type I (T1SS), type II (T2SS), and type V (T5SS) secretion systems, which are among the most conserved elements within the *P. syringae* complex [13]. This conserved pattern contrasts with the structural diversity observed in type III and type VI secretion systems. The type III (T3SS), in *Pss*, is organized as a tripartite pathogenicity island (T-PAI) (Figure 3) and is composed of a central cluster of structural genes (*hrp/hrc*) flanked by two variable regions: the Conserved Effector Locus (CEL) and the Exchangeable Effector Locus (EEL) [14,15].

All genomes displayed the canonical tripartite organization of the pathogenicity island, in full agreement with reference strains, indicating a high level of structural conservation of this system across diverse phylogenetic lineages. The central *hrp/hrc* cluster, which includes 27 virulence-related genes and the regulatory genes *hrpR*, *hrpS,* and *hrpL*, showed nucleotide identity > 95% in all isolates. However, three distinct structural genotypes were identified based on insertions and cluster length, designated T-PAI-1, T-PAI-2, and T-PAI-3 (Figure 3A).

Genotype T-PAI-1 was the most frequent, spanning a 26.5 kb chromosomal region, and was present in 60 isolates from Clusters 1, 4, 6, 9, 10, 11, 12, 14, 15, 16, and 17 (Table 2). It was found in 24 Chilean strains belonging to Clusters 1, 6, 11, 12, 15, and 16. This arrangement contains a 3.3 kb insertion between *hrpV* and *hrpU*, encoding an AAA-family ATPase and an HNH endonuclease. The same genotype is also found in reference genomes pss9097, pssB301D, pssCFBP4215, and pssB728a, and it corresponds to the predominant T-PAI type within the PG2d subgroup (Table S2).

On the other hand, genotype T-PAI-2 was identified in three strains from Cluster 13, a lineage represented exclusively by Chilean isolates (Table S2). This genotype spans a 44.9 kb region. In addition to the insertion between *hrpV* and *hrpU* (as in T-PAI-1), this genotype contains a 21.7 kb insertion between *hrpE* and *hrpF*, encoding twelve ORFs, including a FRG domain protein, a DNA adenine methyltransferase, an 8-oxoguanine glycosylase, a 7-cyano-7-deazaguanine synthase, a PfkB family carbohydrate kinase, a UvrD-like helicase, an ATP-dependent nuclease, a DUF4238 domain protein, a tetratricopeptide repeat protein, and two hypothetical proteins (Figure 3A). This organization was not observed in any reference genome and appears to be exclusive to Chilean isolates.

Finally, genotype T-PAI-3 was detected in 40 isolates belonging to Clusters 2, 3, 4, 5, 6, 7, 8, and 18, with 10 Chilean strains from Clusters 3 and 6 (Table 2). It spans a 23.2 kb region without detectable insertions and was also observed in the reference genomes pssCFBP2118 and psUMAF0158, the latter belonging to subgroup PG2a (Table S2).

Most clusters are composed solely of either T-PAI-1, T-PAI-2, or T-PAI-3 strains (Table 2). In contrast, Clusters 4 and 6 contain strains of both T-PAI-1 and T-PAI-3 types. For a group of 10 strains (Table S2), the configuration was non-determinable (ND) due to inadequate assembly of the draft genomes in the T-PAI regions.

Comparative analysis of the Conserved Effector Locus (CEL) revealed the presence of up to seven open reading frames (ORFs) arranged in a relatively conserved manner across the 113 PG2d genomes analyzed. The CEL typically contains genes encoding several type III secretion system effectors (T3SEs), including *HopM1*, *AvrE1,* and *HopAA1*, together with their corresponding chaperones (*ShcM* and *ShcE*), and in some cases, a putative lytic transglycosylase (SLT) [16]. However, not all of these genes are always present together in every isolate, reflecting some variability in locus composition. The partially conserved order and composition of these genes confirm CEL as a relatively stable genomic region in most *P. syringae* phylogenetic lineages. Its structural conservation and central role in effector delivery underscore its evolutionary importance in pathogen–host interactions [17] (Figure 3B).

In contrast to the highly conserved CEL architecture, the Exchangeable Effector Locus (EEL) showed notable structural and functional variability among the analyzed strains. This region, flanked by the genes *hrpK* and *tRNA^Leu*, is characterized by the presence of T3SEs and accessory proteins that contribute to host specificity and pathogen adaptation to different environments [14] (Figure 3C).

All 113 PG2d genomes strains contained at least one major T3SE within the EEL region (Table S2), with three predominant variants identified: *hopA1* [18], *hopZ3* (classified as *HopBP*/*HopZ*) [19], and a *hopB-like* effector, described as a toxin with a “membrane-targeted effector domain”.

The effector *hopA1* was the most frequently detected T3SE, present in 85/113 genomes. It was widely distributed across many clusters, being found in all members of Clusters 1, 2, 3, 5, 7, 10, 13, 17, and 18, and in some strains from Clusters 8, 12, and 14. Among Chilean isolates, it was present in 26 strains, where two structural variants were distinguished. The long variant, found in all 14 isolates from Cluster 1, included an approximately 5.2 kb insertion between *hopA1* and the *tRNA^Leu* gene, which encodes four additional ORFs of unknown function. This configuration was also observed in the reference genome pss9097. The short variant, identified in 10 genomes from Clusters 3, 12, and 13, lacked this insertion and resembled the organization found in reference strains pssB301D, pssCFBP2118, pssCFBP4215, and psUMAF0158 (Table S2).

The *hopB-like* effector was found in 16/113 strains from Clusters 4, 6, 11, 12, 14, and 15. Nine Chilean isolates carried this effector within their EEL region and were distributed among Clusters 6, 11, 12, 14, and 15 and accompanied by three additional ORFs encoding hypothetical proteins, although no associated chaperone was detected. This genetic architecture matches that of reference strains psyUSA011 and psyUB303, isolated from stream and lake water, respectively. The *hopB-like* effector shared 51.76% identity with HopB1, a canonical effector of the EEL system in *P. syringae* pv. *tomato* DC3000 [20].

Regarding *hopZ3*, 12/113 strains carried this effector, mostly those isolated from *Ph. vulgaris*. Among strains from *Pr. avium*, *hopZ3* was found in three Chilean isolates belonging to Clusters 6 and 16. This hopZ3 effector co-occurred with a chaperone and three additional genes of unknown function. In the strain from Cluster 6, a large insertion (~13.5 kb) between *hopZ3* and the *tRNA^Leu* gene was detected. None of the reference genomes analyzed in this study exhibited a similar EEL organization.

### 2.3. Other Hop Genes Outside of the Pathogenicity Island (PAI)

In addition to the effectors located within the Conserved Effector Locus (CEL) and Exchangeable Effector Locus (EEL), numerous Hop genes were identified outside the pathogenicity island (PAI), scattered throughout the genomes of *P. syringae*, as previously characterized by Dillon et al. [19].

**Figure 4:**
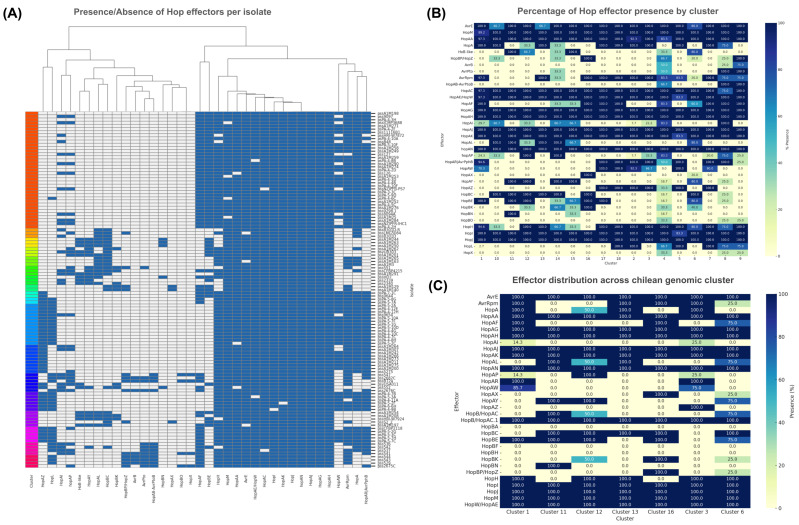
Distribution and diversity of type III effectors (T3SEs) among PG2d lineages. (**A**) Presence/absence heatmap of 36 T3SEs across 113 PG2d genomes. Each column represents an effector; each row represents a strain grouped by PopPUNK cluster. Core effectors (*HopAJ*, *HopJ*, *HopAN*, *HopAG*, *HopAH*, *AvrE*, *HopM*, *HopAA*, *HopAK*) occur in nearly all strains, defining a conserved virulence core. (**B**) Barplots show the mean number of effectors per cluster and their variability, illustrating stable repertoires in Clusters 1, 11, and 12 and higher variability in Clusters 6 and 13. (**C**) YopT-family cysteine proteases (*HopAR*, *HopAY*, *HopAW*) display distinct cluster-specific patterns: *HopAR* + *HopAW* in Clusters 1–3, *HopAY* alone in Clusters 6, 11, 12, and 14, and absence of patterns in Clusters 13 and 16. These differences suggest host-specific adaptation within Chilean *Pss* lineages.

Among the 113 genomes from the PG2d subgroup, a total of 36 different T3SEs were identified (Figure 4A). According to the previously defined cluster, they showed variation in both the number and the identity of effectors (Figure 4B). Cluster 1, the largest, showed an average of 18.95 effectors per strain with low internal variability (standard deviation = 0.66), reflecting a highly conserved effector repertoire. This pattern was also observed in Clusters 11 and 12, each with three strains and no variability (standard deviation = 0.00), indicating a stable functional identity among their members. In contrast, Clusters 10 and 13 had slightly lower averages (16.33 and 16.67 effectors per strain, respectively) and higher standard deviations (0.58), suggesting greater functional variability or recent effector gain/loss events. These differences imply that although most lineages share a conserved core set of effectors, some clusters may be undergoing adaptive diversification, affecting their virulence profiles.

The analysis revealed a set of effectors highly conserved across all strains, regardless of cluster or geographic origin. Five Hop genes were present in 100% of strains: *HopAJ*, *HopJ*, *HopAN*, *HopAG,* and *HopAH*. Additionally, other effectors such as *HopI*, *HopAK*, *HopAC*, *HopAE/HopW*, *AvrE*, *HopAA*, and *HopM* were detected in over 95% of strains, defining a core set of essential T3SS effectors in *Pss*. Some of these effectors have been reclassified as helper proteins (secreted through the T3SS but not translocated into the host cytoplasm), while others may not be secreted at all [21,22]. *HopAN*, *HopAC*, *HopJ*, *HopAJ,* and *HopAK* belong to this group.

On the other hand, several effectors were identified with distributions restricted to specific hosts or genomic clusters, suggesting possible host adaptation events. The effector *AvrB* was exclusive to seven strains isolated from *P. vulgaris* and was absent in all strains from *Prunus* or other hosts, suggesting functional specialization toward legumes. In contrast, *HopAW* was found only in strains isolated from *Prunus* species, indicating a possible role in pathogenic adaptation to these fruit trees. *HopAW* was present in multiple lineages, including Clusters 1, 2, 3, 5, 7, 17, and 18.

Focusing specifically on the Chilean isolates, the general patterns described for the PG2d subgroup remain largely consistent (Figure 4C). A set of 12 effectors—*AvrE*, *HopAJ*, *HopJ*, *HopI*, *HopM*, *HopAA*, *HopAH*, *HopAG*, *HopAK*, *HopAN*, *HopW/HopAE,* and *HopB/HopAC*—was detected in 100% of the Chilean genomes analyzed, reinforcing their role as core components of the *Pss* virulence repertoire. This group defines a highly conserved functional core, regardless of genomic cluster.

Nevertheless, some effectors showed variable distributions across clusters. For instance, *HopA* was found in all isolates from Clusters 1 and 13 but was absent in Clusters 11, 14, and 16, and detected in only 50% of Cluster 12 strains. Similarly, *AvrRpm* was present in all members of Clusters 1, 3, and 13 but absent in Clusters 11, 12, and 15, and it was only partially present in Cluster 6 (25%).

Cluster 1, which includes the highest number of Chilean isolates, exhibited a highly homogeneous effector repertoire. In contrast, Clusters 6 and 16 showed more variable profiles, suggesting potential effector gain/loss events or ongoing local adaptation processes.

Within this effector group, the C58 YopT-type cysteine protease family was identified, previously described as a set of candidate effectors associated with virulence in *Prunus* species [3]. Three representatives of this family were detected among the Chilean isolates: *HopAR* (also known as *AvrPphB*), *HopAY*, and *HopAW*. A consistent distribution pattern was observed: strains carrying *HopAW* also simultaneously harbored *HopAR*, as seen in isolates from Clusters 1 and 3. In contrast, strains from Clusters 6, 11, 12, and 14 contained only *HopAY*, while Clusters 13 and 16 lacked effectors from this family.

These findings indicate that, alongside a highly conserved core effector set, differences in effector presence across genomic clusters reflect active evolutionary processes, likely shaped by host–pathogen interactions and specific agro-environmental pressures within Chilean sweet cherry orchards.

### 2.4. Type VI Secretion System (T6SS) in P. syringae

The type VI secretion system (T6SS) is a nanomolecular complex present in many Gram-negative bacteria, generally composed of around 13 core components, with or without accessory proteins, depending on the species. It functions as a contractile needle capable of injecting toxic effectors directly into target cells, whether they are competing bacteria or host eukaryotic cells [13]. This system plays a fundamental role in both interspecies competition and bacterial virulence, promoting colonization, survival, and adaptation in complex environments, such as the rhizosphere or plant tissues.

T6SS is divided into four classes: i, ii, iii, and iv. Type i is the most classical and widely studied, and it is further subclassified into i1, i2, i3, i4a, i4b, and i5 [23].

**Figure 5:**
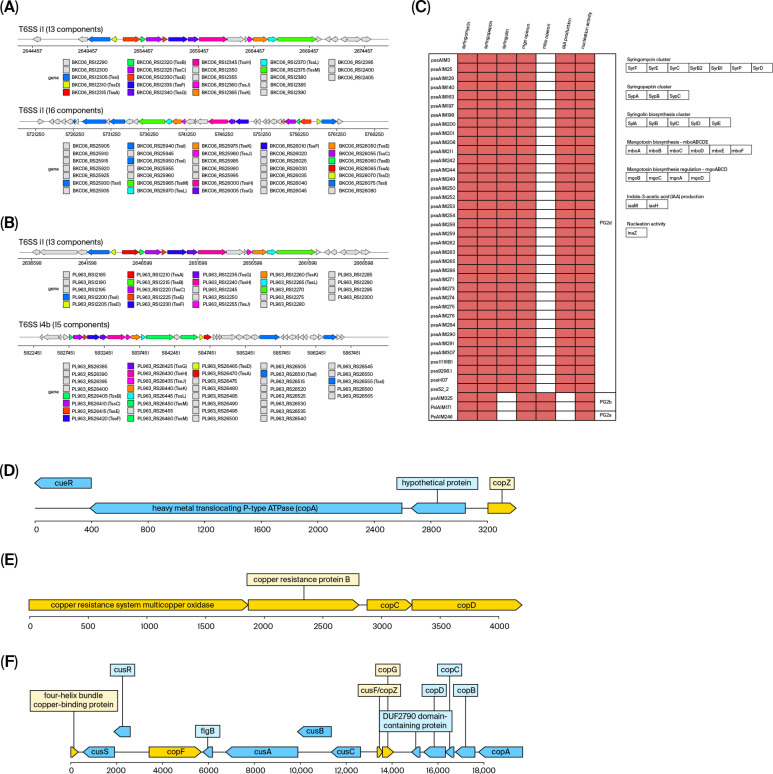
Type VI secretion systems, toxin-related genes, and copper resistance operons in Chilean *Pseudomonas syringae* pv. *syringae*. (**A**,**B**) Structural diversity of type VI secretion system (T6SS) gene clusters among PG2d genomes. Two major configurations were identified: a dual i1-type cluster (present in Cluster 1 and reference strains pss9097, pssB48, pss9644) and a combined i1/i4a-type arrangement in other lineages. Colored blocks represent core structural and accessory components, highlighting the duplication of the i1 system in several Chilean isolates. (**C**) Heatmap showing the presence or absence of phytotoxin biosynthetic clusters among PG2d strains. All Chilean isolates harbor complete operons for syringomycin, syringopeptin, and syringolin production, while genes for coronatine, tabtoxin, and phaseolotoxin are absent. The *mgoABCD* regulatory operon is conserved, but the *mboABCDEF* biosynthetic operon is missing, indicating loss of mangotoxin synthesis. This pattern, shared with reference PG2 strains, supports functional specialization in the toxigenic repertoire of Chilean *Pss*. (**D**–**F**) Representative architectures of copper homeostasis and resistance operons. (**F**) The extended *cusCBA* + *copABCD* operon, detected in Clusters 1 and 16, combines cus and cop systems together with regulatory (*cusR*/*S*, *copG*) and chaperone (*cusF*/*copZ*) genes, suggesting synergistic resistance to Cu^+^/Cu^2+^ ions and reflecting adaptation to copper-intensive agricultural environments.

The presence and diversity of T6SS gene clusters within the PG2d phylogroup, including 38 Chilean strains and nine complete reference genomes from the same group, was evaluated. The analysis revealed three distinct T6SS clusters: two corresponding to subtype i1 (one composed of 13 components and the other of 16) (Figure 5A) and one corresponding to subtype i4a (composed of 13 components) (Figure 5B). Remarkably, all genomes analyzed contained two T6SS clusters, confirming that the dual presence of these systems is a conserved feature within this phylogroup. Specifically, Chilean strains belonging to Cluster 1 carried two i1-type clusters, similar to the reference strains pss9097, pss9644, and pssB48. Interestingly, Cluster 17 displayed the same configuration (Table 2). In contrast, Chilean isolates from the other clusters presented one i1-type cluster (the one with 13 components) and one i4a-type cluster, a pattern matching the reference genomes psyUB303, psyUSA011, pssB301D, pssCFBP2118, and pssCFBP4215.

This differential distribution suggests potential functional specialization or redundancy in secretion machinery, possibly associated with different ecological niches or strategies for interacting with hosts and other microorganisms.

### 2.5. Production of Phytotoxins

Various strains of the *P. syringae* complex are capable of producing a wide range of phytotoxins, including coronatine, syringomycin, syringopeptin, syringolin, mangotoxin, tabtoxin, and phaseolotoxin, which play key roles in symptom development, suppression of host defenses, and facilitation of tissue colonization [24].

In this study, the presence of biosynthetic gene clusters associated with these toxins was evaluated in Chilean isolates (Figure 5C). All analyzed genomes contained a complete cluster for syringomycin and syringopeptin biosynthesis, comprising the genes *syrF*, *syrE*, *syrC*, *syrB2*, *syrB1*, *syrP*, *syrD*, *sypA*, *sypB,* and *sypC*, confirming their role as central virulence determinants in these lineages. Similarly, the syringolin biosynthetic cluster, composed of *sylA*, *sylB*, *sylC*, *sylD,* and *sylE*, was consistently detected in all Chilean genomes (Figure 5C).

This genetic configuration was also observed in the nine complete reference genomes of the PG2d group, supporting that the biosynthetic capacity for syringomycin and syringopeptin is a typical and conserved feature within this phylogenetic group [25].

Interestingly, although the mangotoxin regulatory operon (*mgoABCD*) [26] was identified in the Chilean isolates as well as in the nine PG2d reference genomes, the biosynthetic operon *mboABCDEF* [27] was absent, suggesting that these strains may have lost the ability to produce this toxin. This feature has also been observed in strains belonging to the PG2a phylogroup, indicating a possible shared evolutionary pattern within the PG2 group [28]. On the other hand, no genes related to the production of coronatine, tabtoxin, or phaseolotoxin were detected, indicating that these compounds are not part of the predominant toxigenic repertoire of the Chilean isolates and PG2 strains more broadly (Figure 5C).

### 2.6. Other Metabolic Virulence-Associated Factors

Other metabolic factors associated with bacterial virulence, such as IAA production and the presence of ice nucleation protein, were analyzed. All PG2d strains carried the *iaaM* (tryptophan monooxygenase) and *iaaH* (indoleacetamide hydrolase) genes, which constitute the core enzymatic machinery for the biosynthesis of indole-3-acetic acid (IAA) from L-tryptophan, an auxin capable of modulating host hormonal responses and promoting infection (Figure 5C). These genes were also detected in the complete reference genomes of PG2d, confirming that IAA production is a conserved feature within this phylogenetic subgroup. Notably absent in subgroups PG2a, PG2b, and PG2c, these genes likely play a specialized role in their plant host infection process, unique to PG2d (Table S3). However, given that the PG2c subgroup is represented by only two genomes in this analysis, its absence there should be interpreted with caution; the finding is statistically robust for the well-sampled PG2a (*n* = 26), PG2b (*n* = 52), and PG2d (*n* = 113) subgroups.

The presence of the *inaZ* gene, which encodes an ice nucleation protein widely characterized in *P. syringae*, was also consistently detected. This protein enables the formation of ice crystals at temperatures near 0 °C, causing frost injuries that facilitate pathogen entry into plant tissues. Ice nucleation ability is a hallmark of the *P. syringae* complex and has been described as a key virulence factor under cold environmental conditions [10]. The conservation of *inaZ* across all Chilean isolates suggests a possible adaptation to temperate climates and highlights its potential role during the early stages, at the end of winter, of infection under field conditions. This gene was present in all 152 *P. syringae* genomes analyzed belonging to the PG2 phylogenetic group.

### 2.7. Repertoire of Copper Resistance Genes in Pss

The analysis of genomic regions associated with copper homeostasis and resistance revealed the presence of different systems of copper control in the Chilean isolates. This analysis also included the plasmids that were detected in 10 isolates (five from Cluster 1, three from Cluster 11, and two from Cluster 16). It is important to note that the absence of plasmid predictions in the remaining isolates does not necessarily indicate their absence in vivo, as some may not have been captured due to sequencing and assembly limitations. The same criterion should be applied for downloaded international strains that only provide the assembled genomes without plasmids.

All strains shared a conserved *cueR–copA–copZ* module (Figure 5D), also known as the *cueAR* operon [29]. This module represents a basic mechanism of intracellular copper resistance, where CueR acts as a transcriptional sensor regulating *copA*, responsible for Cu^+^ efflux from the cytoplasm. The consistent presence of *copZ*, likely functioning as a periplasmic chaperone, indicates a conserved functional system adapted to the agricultural environment of cherry orchards.

The classic *copABCD* operon was identified in isolates from Cluster 3 (Figure 5E). This configuration suggests a bidirectional, multi-phase copper management system in which *copC* and *copD* may participate in copper uptake under scarcity, while *copA* and *copB* mediate efflux under toxic conditions [30]. In addition, this operon was also observed in isolates from Cluster 10, which were obtained from *Pyr*. *communis*. Some isolates from other clusters harbored only *copA* and *copB*, without *copC* or *copD*. This truncated version suggests simplified functional variants, possibly sufficient to confer a basal level of copper resistance, though likely less efficient than complete operons.

Remarkably, the presence of an extended operon that consists of the *cusCBA* and *copABCD* systems was found in a specific subset of Chilean isolates, including regulatory, transport, and copper-binding elements. The observed gene operon architecture was as follows, four-helix bundle copper-binding protein–*cusS*–*cusR*–*copF*–*cusA*–*cusB*–*cusC*–*cusF/copZ*–*copG*–*copD*–*copC*–*copB*–*copA* (Figure 5F), similar to the structure described by Gutiérrez-Barranquero et al. [31]. This operon was detected in all 14 Chilean strains from Cluster 1. Ten strains carry it on the chromosome, while the other four have it on plasmids. It is also present in two reference genomes: pss9644 and pssB48. Also, the two plasmids detected in Cluster 16 contained complete copper resistance systems, including structural and regulatory genes from the *copABCD* and *cusCBA* operons. Moreover, the presence of this operon was also observed in isolates from Clusters 2, 5, and 17, indicating that the distribution of this copper resistance determinant is broader within the PG2d phylogroup than initially expected.

The combination of *cus* and *cop* systems, along with regulatory proteins (*copG*, *cusR*) and copper chaperones (*copZ/cusF*), suggests a synergistic and highly specialized configuration for the expulsion of Cu^+^ and Cu^2+^ ions [31]. In this context, *copF* and *copA* encode P-type ATPases for active copper efflux, *cusCBA* forms an RND-type efflux complex, and *copZ/cusF* facilitate the safe periplasmic transfer of copper ions. Additionally, the presence of the ribbon–helix–helix transcriptional regulator *copG* and the two-component system *cusS–cusR* indicates tight gene expression control in response to toxic copper concentrations [32].

### 2.8. Pangenome Analysis and Orthogroup Distribution

**Figure 6:**
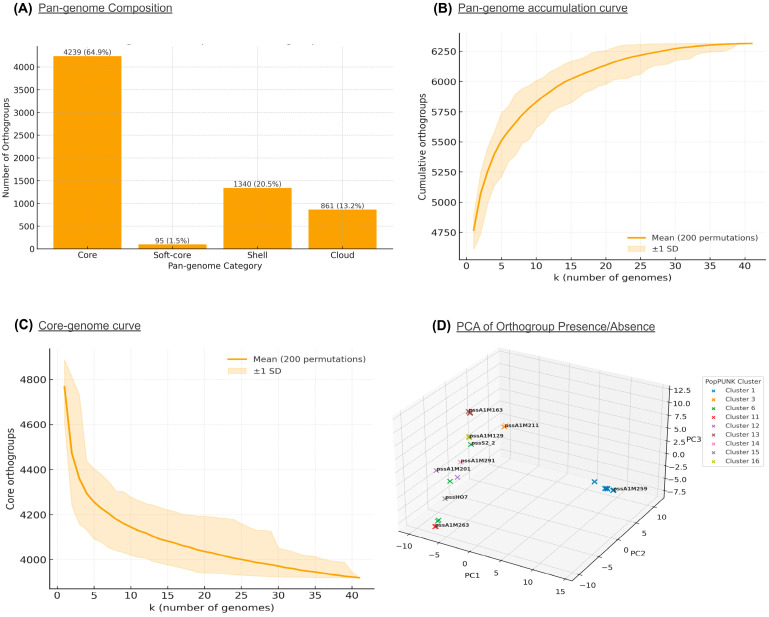
Pangenome composition and diversity of Chilean *Pseudomonas syringae* pv. *syringae* isolates. (**A**) Partition of the 6535 orthogroups inferred with OrthoFinder into core (64.9%), soft-core, shell, and cloud categories. (**B**,**C**) Pangenome accumulation curve fitted to Heaps’ law showing α = 1.87 (R^2^ = 0.83), indicating a closed pangenome under current sampling. (**D**) Principal Component Analysis (PCA) of orthogroup presence/absence across PopPUNK clusters reveals variable intra-cluster genomic diversity, with low variance in Clusters 1 and 3 (stable lineages) and high variance in Cluster 6 (indicative of recent diversification or horizontal gene transfer). Together, these results indicate a largely conserved genomic backbone with adaptive diversification among local Chilean lineages.

To characterize the genomic diversity and conserved gene content of the 38 *Pss* isolates that fall within the PG2d phylogroup, collected from cherry orchards across different regions of Chile, a pangenome analysis was conducted based on orthogroup inference using OrthoFinder. In total, 6535 orthogroups were identified, of which 4239 (64.9%) comprised the core genome, defined as genes present in all strains. Within this conserved core, 3832 orthogroups corresponded to single-copy genes, making them ideal candidates for phylogenomic and evolutionary studies. Beyond the core, the accessory genome included 95 soft-core orthogroups (present in ≥95% but <100% of strains), 1340 shell orthogroups (present in 15–95% of strains), and 861 cloud orthogroups (present in ≤15% of strains), highlighting considerable diversity within the accessory genome (Figure 6A).

Further, analysis of rarefaction curves based on the orthogroup presence/absence matrix [33] indicates that the pangenome accumulation curve reaches a plateau after incorporating all 38 isolates (Figure 6B,C). According to Heaps’ law, this curve yields α = 1.872 (95% CI: 1.815–1.969; R^2^ = 0.825) per new orthogroups per added genome at k = 38, which is consistent with a closed pangenome under our sampling. Together with the high proportion of the core genome (4239 orthogroups; 64.9%), these results suggest that incorporating additional Chilean isolates is unlikely to substantially expand the catalog of gene families.

To assess variation among PopPUNK-defined lineages, a binary presence/absence matrix of orthogroups by cluster membership was constructed. An orthogroup was considered cluster-exclusive if present in 100% of its members and absent in all others. Based on this criterion, 154 exclusive orthogroups were identified in Cluster 1, 91 in Cluster 3, 82 in Cluster 16, 65 in Cluster 13, 52 in Cluster 11, and 36 in Cluster 12. No exclusive orthogroups were found in Clusters 6, 14, and 15. In the latter two, this was expected given that each is represented by a single Chilean strain, making it impossible to define internally shared orthogroups.

To evaluate the degree of genomic variability within each cluster, a Principal Component Analysis (PCA) was performed on the binary orthogroup presence/absence matrix. Each isolate was represented in a reduced three-dimensional space, and clusters were assessed based on the variance of their principal components (Figure 6D). The analysis revealed that Cluster 6 exhibited the highest internal diversity (mean variance = 15.2), despite being composed of only four Chilean isolates, which could reflect recent microevolutionary processes or horizontal gene acquisition events. In contrast, Clusters 1 and 3, which included the majority of Chilean isolates (*n* = 13 and 8, respectively), displayed substantially lower intra-cluster variance (0.39 and 0.02), indicating greater homogeneity in gene content. These findings suggest that while some lineages exhibit stable and conserved genomic structures, others may be undergoing accelerated diversification processes.

## 3. Discussion

This study provides a comprehensive genomic characterization of *Pseudomonas syringae* pv. *syringae* isolates collected from sweet cherry orchards in Chile, enabling their contextualization within the PG2 phylogenetic group on a global scale. The results reveal a clear predominance of the PG2d subgroup among Chilean strains, with a population structure defined by at least 18 genomic clusters, several of which appear to be exclusive to Chile. This pattern suggests local diversification processes, potentially driven by specific agroclimatic conditions or adaptation to *Pr. avium* cultivars.

The predominant grouping of Chilean strains in PG2d is consistent with studies from other temperate regions, where this subgroup also dominates in sweet cherry, pear, and legume crops [17,34]. However, the identification of exclusive clusters in Chile supports the hypothesis of geographic isolation and local selective pressures shaping the genomic evolution of these lineages. Factors such as intensive copper use, soil and climate conditions in fruit-growing valleys, or region-specific agricultural practices may be acting as drivers of this diversification.

The pangenome analysis revealed notable plasticity in the accessory gene content between lineages, despite the presence of a common core of over 4100 orthogroups. The identification of cluster-specific orthogroups supports the idea of adaptive specialization, potentially related to host–pathogen interactions, heavy metal tolerance, or microbial competition. In particular, the low intra-cluster diversity observed in Clusters 1 and 3 may reflect recent clonal expansion of locally adapted lineages, while the high variability in Cluster 6 suggests more dynamic horizontal gene acquisition, likely facilitated by interactions with other bacteria in the plant microbiome.

The secretion system repertoire showed a hierarchical conservation pattern: while T1SS, T2SS, and T5SS were ubiquitously present and conserved, T3SS and T6SS displayed greater structural variability and differential distribution among clusters. It is known that T1SS, T2SS, and T5SS systems primarily participate in the secretion of enzymes and proteins into the extracellular environment and play complementary roles to the specialized T3SS and T6SS systems [13,35]. T1SS, composed of three main components, enables direct export of toxins and proteases from the cytoplasm to the extracellular space [36]. T2SS operates via a two-step mechanism and is associated with the secretion of hydrolytic enzymes that degrade components of the host plant cell wall [37]. Meanwhile, T5SS, also known as the autotransporter system, facilitates the secretion of adhesins and other proteins involved in surface colonization [38]. The universal conservation of these systems across all analyzed genomes underscores their functional importance in the general biology of *P. syringae*, beyond their specific role as a phytopathogen.

In the case of T3SS, three structural genotypes of the T-PAI pathogenicity island were identified, one of which (T-PAI-2) is exclusive to Chilean isolates and contains insertions that may encode new regulatory or accessory effector functions. This plasticity, together with the variability observed in the EEL *locus*, illustrates the evolutionary dynamics of T3SS effector repertoires. Some, such as *HopAW* and *HopAX*, showed restricted distribution in strains isolated from *Prunus*, suggesting host–pathogen co-evolution. In contrast, *AvrB* was exclusive to strains from *Ph. vulgaris*, reflecting functional specialization toward legumes, as reported in other pathovars.

The distribution of effectors from the C58 YopT-type cysteine protease family observed in our Chilean isolates is consistent with findings from other cherry pathogens, where HopAR1 has been identified as a central candidate associated with virulence in *Prunus*-pathogenic clades [39]. In our study, the co-occurrence of *HopAR* and *HopAW* in the main Chilean clusters (1 and 3) reinforces the hypothesis that these proteases may play a relevant role in adaptation to *Pr. avium*. By contrast, other lineages (Clusters 6, 11, 12, and 14) carried only *HopAY*, while Clusters 13 and 16 lacked effectors from this family, suggesting divergent evolutionary trajectories and potential differences in host target repertoires.

As described in other *P. syringae* pathogens, the presence of multiple *YopT* family representatives within the same genome, particularly *HopAR* and *HopAW*, may indicate partial functional redundancy or complementary specialization, pointing to a common selective pressure to modulate basal immunity responses in cherry. Although HopAR has been characterized as a cysteine protease capable of cleaving key cytoplasmic kinases in *Arabidopsis* and barley [40,41], its exact role in cherry remains undefined and requires experimental validation through the identification of interacting host proteins. In this context, the diversity of combinations observed among *HopAR*, *HopAY,* and *HopAW* in Chilean isolates may reflect distinct modes of local adaptation and potential differences in host specificity.

Regarding the type VI secretion system (T6SS), two main configurations were detected among Chilean isolates: one with two copies of subtype i1 (predominant in Cluster 1), and another combining subtypes i1 and i4a (found in other clusters). This distribution, also observed in reference strains, suggests possible functional redundancy or ecological specialization in competitive environments, such as the plant microbiome. In particular, duplication of the i1-type T6SS in Cluster 1 may confer an adaptive advantage in intensive agricultural contexts like Chilean cherry orchards by enhancing competitive exclusion and environmental persistence [42].

The analysis of virulence-associated metabolic factors revealed strong conservation of the syringomycin, syringopeptin, and syringolin biosynthetic clusters, underscoring their central role in PG2d pathogenicity. In contrast, no complete operons for coronatine, tabtoxin, or phaseolotoxin production were detected. Additionally, although the mangotoxin regulatory operon (*mgoABCD*) was identified, the absence of the biosynthetic cluster (*mboABCDEF*) suggests functional loss or alternative regulation not yet characterized. This pattern, which has also been reported in PG2a strains, may reflect an evolutionary trend within PG2 lineages, where the loss of mangotoxin production could represent an adaptation to specific hosts or ecological niches.

The exclusive presence of *iaaM* and *iaaH* genes, associated with indole-3-acetic acid (IAA) synthesis, in the PG2d subgroup, and their absence in PG2a, PG2b, and PG2c, suggests a specialized mode of host interaction that modulates plant hormonal responses. However, the limited number of available PG2c genomes (*n* = 2) precludes a definitive conclusion for this subgroup. Based on the symptoms that *Pss* produces in *Prunus* plants, this interaction likely involves the suppression of host defense responses, as high IAA levels can inhibit SA-mediated defenses, thereby increasing plant susceptibility [43].

The persistent presence of the *inaZ* (ice nucleation) gene in all the PG2 group (PG2a, PG2b, PG2c, and PG2d) indicates that ice nucleation is a key functional trait of this group, facilitating entry into plant tissues under cold conditions.

In terms of copper resistance, multiple operonic configurations were identified, ranging from truncated versions to extended operons combining *cop* and *cus* systems. The latter configuration, detected in several Cluster 1 strains, suggests adaptation to agricultural environments where copper is routinely applied as a bactericide. The universal presence of the *cueR–copA–copZ* module in all isolates points to a highly conserved basal resistance mechanism essential for bacterial survival under metal stress. Additionally, plasmid analysis revealed that extrachromosomal elements play a crucial role in the adaptation of Chilean *Pss* populations. Notably, plasmids found in Clusters 1 and 16 harbor complete copper resistance operons (*copABCD* and *cusCBA*), suggesting that these replicons not only complement but may also substitute for missing chromosomal systems. This pattern was consistent with the absence of the *cusCBA* operon in the chromosomes of those strains, indicating that copper resistance in these lineages depends exclusively on plasmid content. Together, these findings not only enhance our understanding of the genomic diversity of *Pss* in Chile but also illustrate how ecological factors, agricultural practices, and host–pathogen dynamics shape the genomic and functional evolution of phytopathogenic populations. The detection of exclusive clusters, secretion system variants, differential toxin profiles, and both chromosomal and plasmid-borne resistance operons reinforces the hypothesis of local adaptation. The dual role of plasmids as resistance elements and vehicles of horizontal gene transfer suggests that their genomic surveillance may be key in future phytosanitary management strategies.

Using a core-genome phylogeny of 197 genomes representing the entire PG2 lineage, the evolutionary origin of the PG2d phylogroup was analyzed by performing probabilistic ancestral reconstruction of gain and loss events on 1772 shell gene families using GLOOME [44]. The PG2d subphylogroup formed a fully supported, monophyletic clade (113/113 genomes). Its ancestral branch showed a marked elevation in both gene gains (1671) and losses (1592), indicative of a period of extensive genomic remodeling at the origin of this lineage. In contrast, PG2a was not monophyletic, and its ancestral gene flux could not be traced to a single branch. PG2b exhibited a pronounced signal of gene flux within its main clade, although this pattern was complicated by a few outlier genomes. For PG2c, the signal was clear but limited by the small sample size. The marked and simultaneous increase in gene gains and losses specific to the PG2d ancestor is consistent with a model of major adaptive transition, in which the acquisition of novel functions is followed by genome streamlining, a classic hallmark of ecological specialization. Accordingly, unlike the more heterogeneous evolutionary histories inferred for PG2a and PG2b, the cohesive signal observed in PG2d allows its distinctive gene content to be attributed to a single ancestral shift. This strongly supports the hypothesis of a shared adaptive trajectory, potentially linked to specialization to a particular host or environmental niche.

Despite the strong phylogenetic cohesion of PG2d at the core-genome level, PopPUNK identified multiple genomic clusters that reflect recent diversification patterns driven primarily by gene content variation. Cluster 1, the largest and most diverse group, comprises strains isolated from multiple Prunus hosts and a broad geographic range, suggesting a widely distributed and host generalist lineage within PG2d. In contrast, Clusters 3 and 6 are enriched in isolates from *Pr. avium*, predominantly from Chile, indicating more localized diversification and potential adaptation to regional or agricultural niches. Notably, these PopPUNK clusters do not necessarily correspond to strictly monophyletic groups in the core-genome phylogeny, underscoring a decoupling between phylogenetic relationships and gene content similarity. This pattern is consistent with ongoing reshuffling of shell gene families following the ancestral genomic remodeling event inferred at the origin of PG2d, providing a mechanistic link between the deep evolutionary signal detected by GLOOME and the contemporary population structure revealed by PopPUNK

### Limitations and Future Perspectives

This study provides a comprehensive genomic characterization of the dominant Pss populations affecting Chilean sweet cherry orchards. However, some limitations should be considered. The phylogenetic analysis reveals an uneven distribution of isolates across subclades, with PG2d being highly prevalent (*n* = 113), while PG2a (*n* = 26), PG2b (*n* = 52), and PG2c (*n* = 2) are represented by fewer genomes. Consequently, conclusions about the absence of specific genes in minor groups should be interpreted with caution, as they may reflect sampling constraints rather than true biological signatures. Future studies should prioritize expanding the collection to include more isolates from these underrepresented phylogenetic groups, both within Chile and from other geographical regions, to confirm their genetic repertoire and ecological significance.

Despite the depth of the genomic analysis, this study is based exclusively on in silico information. The integration of functional assays and in planta pathogenicity experiments will be essential to validate the expression and biological role of the virulence and resistance genes identified. Likewise, expanding the sampling to other fruit-growing regions of Chile and to alternative hosts could reveal new lineages and provide insights into additional evolutionary trajectories.

Future studies combining functional genomics, bacterial competition assays, and environmental monitoring will be fundamental to understanding the mechanisms underlying local adaptation, population dynamics, and the epidemiological impact of *Pss* in Chilean fruit production. Furthermore, the identification of dominant, locally adapted clusters (e.g., Clusters 1 and 3) provides a clear target for developing cluster-specific molecular detection tools. Ultimately, characterizing the effector repertoire and host susceptibility mechanisms associated with these predominant lineages could inform the development of resistant cherry cultivars.

## 4. Materials and Methods

### 4.1. Bacterial Strains and Genome Sequencing

A total of 41 *P. syringae* strains were analyzed in this study. All isolates were obtained from symptomatic tissues of sweet cherry trees (*Prunus avium* L.) displaying typical symptoms of bacterial canker. Sampling was conducted in the main cherry-producing regions of Chile (Table 1). Of these, 31 strains belonged to the *Pseudomonas* strain collection of INIA-Rayentué, preserved in the Chilean Collection of Microbial Genetic Resources (CChRGM), as previously described by Correa et al. [7]. Four additional strains were provided by the Phytopathology Laboratory at the University of Chile. Six new strains were recently isolated for this study from fruits, leaves, and necrotic buds with symptoms.

Genomic DNA was extracted and sequenced following the protocols described by [7]. Assembled genomes were arranged into pseudochromosomes using Ragout v2.3 [45] with default parameters and publicly available *P. syringae* reference genomes as guides (Table S1). Genomes were annotated with Bakta v1.11 [46] to ensure consistent gene prediction and functional assignment. Sequencing data were deposited in NCBI under Bio Project accession number PRJNA750090.

### 4.2. Comparative Genomics and Functional Annotation

To contextualize Chilean isolates at a global scale, 152 public *P. syringae* genomes belonging to the PG2 phylogenetic group were obtained from NCBI and the Pseudomonas Genome Database [47] (Table S1). These included strains isolated from *Prunus* spp. and other plant hosts across diverse geographic regions. Additionally, eight PG3 group genomes isolated from sweet cherry trees were included as outgroups.

Average Nucleotide Identity (ANI) was calculated using Skani v0.1.3 [48], and pairwise distance matrices were used to perform hierarchical clustering, generating a dendrogram to evaluate genomic relationships. Population structure was determined using PopPUNK v2.4.0 [12], which compares core and accessory genome distances via variable-length k-mers to define lineages.

Pangenome analysis was performed using OrthoFinder v2.5.5 [49], employing protein sequences annotated with Bakta v1.11. This allowed identification of core, shell, and cloud gene fractions and detection of orthogroups specific to PopPUNK-defined clusters, defined as genes present in all members of a lineage and absent in others. Pangenome openness was further evaluated by generating rarefaction curves and fitting Heaps’ law to the average number of novel orthogroups contributed per genome. These analyses were implemented through a custom Python v3.10 script, following the approach described in [33], where the parameter α\alphaα of the power law indicates whether the pangenome is open (α < 1) or closed (α > 1). The evolutionary origin of the phylogroups were evaluate with GLOOME v2.0 [44] using shell gene families identified by PopPUNK v2.4.0.

All predicted proteins were functionally annotated using eggNOG-mapper v2.1.7 [50], providing KEGG pathway assignments, GO terms, and COG classifications for subsequent comparative analyses.

### 4.3. Identification of Virulence and Copper Resistance Factors

To investigate the pathogenic potential and adaptive traits of Chilean isolates, all genomes were screened for previously described virulence and copper resistance determinants in *P. syringae* [51,52]. The presence of major bacterial secretion systems and associated effectors was evaluated, including type I (T1SS), type II (T2SS), type III (T3SS), type V (T5SS), and type VI (T6SS) systems [13,53]. Specifically, the SecReT6 platform [42] was used to investigate the presence and diversity of T6SS gene clusters. Conserved virulence factors in the *P. syringae* complex were also analyzed, such as genes related to ice nucleation, phytohormone (auxin) synthesis, and phytotoxin biosynthesis [24,54,55]. Genes associated with copper resistance were examined, with emphasis on detecting the *copABCD* and *cusCFBA* operons and their regulatory elements (*copR/copS*, *cusR/cusS*, *cueR*) [51]. The presence of these elements was also evaluated in plasmids using the tool Platon v1.6.0 [56]. This software infers plasmids from short-read assemblies based on replicon distribution scores derived from protein sequences.

For these searches, HMM profiles were built from homologous sequences derived from reference genomes, and hmmsearch was applied with stringent thresholds: minimum coverage of 50% and e-value ≤ 1 × 10^−40^. The genomic context of each finding was manually reviewed to validate operon structure, functional co-localization, and completeness of each cluster.

All detected virulence and resistance genes were assigned to the orthogroups generated by OrthoFinder, enabling systematic comparisons between genomes and PopPUNK-defined lineages. This integrative approach facilitated the identification of lineage-specific patterns and the evaluation of diversity and conservation of key elements associated with pathogenicity in the Chilean population of *P. syringae* pv. *syringae*.

## Figures and Tables

**Table 1:** *Pseudomonas syringae* pv. *syringae* strains isolated from *Prunus avium* (sweet cherry) orchards in Chile. Information includes strain code, host tissue, geographic origin, GPS coordinates, species/pathovar, RefSeq accession number at NCBI, bibliographic reference, and assigned phylogroup.

Strain	Host Tissue	Geographic Origin in Chile	GPS Coordenates	Species/Pathovar	RefSeq Assembly Accession	Reference	Phylogroup
pssA1M3	Leaf wash	Ñuble	−36.595383, −72.088307	*P. syringae* pv. *syringae*	GCF_022560185.1	[7]	PG2d
pssA1M25	Cankerous wood	Los Lagos	−40.939380, −72.875670	*P. syringae* pv. *syringae*	GCF_022509985.1	[7]	PG2d
pssA1M129	Macerated buds	O’Higgins	−34.332262, −70.876175	*P. syringae* pv. *syringae*	GCF_022510065.1	[7]	PG2d
pssA1M140	Macerated buds	O’Higgins	−34.684579, −70.854987	*P. syringae* pv. *syringae*	GCF_030504425.1	[7]	PG2d
pssA1M163	Macerated buds	O’Higgins	−34.372358, −71.073199	*P. syringae* pv. *syringae*	GCF_022510045.1	[7]	PG2d
psA1M171	Macerated buds	O’Higgins	−40,378504; −73,042016	*Pseudomonas syringae*	GCF_022510085.1	[7]	PG2b
pssA1M197	Cherry fruit	Araucanía	−38.339633, −72.716477	*P. syringae* pv. *syringae*	GCF_022510105.1	[7]	PG2d
pssA1M198	Cherry fruit	Maule	−35.922623, −71.778490	*P. syringae* pv. *syringae*	GCF_022510135.1	[7]	PG2d
pssA1M200	Unknown	Ñuble	−36.689146, −72.426716	*P. syringae* pv. *syringae*	GCF_022510125.1	[7]	PG2d
pssA1M201	Unknown	Los Lagos	−40.330155; −73.1813930	*P. syringae* pv. *syringae*	GCF_022510165.1	[7]	PG2d
pssA1M208	Unknown	Ñuble	−36.634185, −71.8999459	*P. syringae* pv. *syringae*	GCF_022510185.1	[7]	PG2d
pssA1M211	Unknown	Ñuble	−36.689525, −72.426652	*P. syringae* pv. *syringae*	GCF_022510205.1	[7]	PG2d
pssA1M242	Unknown	Ñuble	−36.689146, −72.426716	*P. syringae* pv. *syringae*	GCF_022510225.1	[7]	PG2d
pssA1M244	Cankerous wood	Los Ríos	−39.635661, −73.138562	*P. syringae* pv. *syringae*	GCF_022510265.1	[7]	PG2d
psA1M246	Unknown	Unknown	Unknown	*Pseudomonas syringae*	GCF_022513625.1	[7]	PG2a
pssA1M249	Cankerous wood	Maule	−35.925665, −71.777488	*P. syringae* pv. *syringae*	GCF_022513665.1	[7]	PG2d
pssA1M250	Leaf lesion	Los Lagos	−40.810499, −73.226783	*P. syringae* pv. *syringae*	GCF_022513725.1	[7]	PG2d
pssA1M252	Cankerous wood	Maule	−35.925665, −71.777488	*P. syringae* pv. *syringae*	GCF_022513685.1	[7]	PG2d
pssA1M253	Cankerous wood	Los Lagos	−40.764471, −72.940451	*P. syringae* pv. *syringae*	GCF_022513735.1	[7]	PG2d
pssA1M254	Cankerous wood	Maule	−35.924664, −71.779454	*P. syringae* pv. *syringae*	GCF_022513705.1	[7]	PG2d
pssA1M258	Cankerous wood	Araucanía	−38.341199, −72.716877	*P. syringae* pv. *syringae*	GCF_022513765.1	[7]	PG2d
pssA1M259	Cankerous wood	Ñuble	−36.446398, −71.813875	*P. syringae* pv. *syringae*	GCF_022510285.1	[7]	PG2d
pssA1M262	Cankerous wood	Los Lagos	−40.532192, −73.072365	*P. syringae* pv. *syringae*	GCF_022510235.1	[7]	PG2d
pssA1M263	Leaf lesion	Los Lagos	−40.532535, −73.073120	*P. syringae* pv. *syringae*	GCF_022510305.1	[7]	PG2d
pssA1M265	Macerated buds	Ñuble	−36.448562, −71.814961	*P. syringae* pv. *syringae*	GCF_022513785.1	[7]	PG2d
pssA1M266	Macerated buds	Ñuble	−36.449630, −71.815074	*P. syringae* pv. *syringae*	GCF_022513815.1	[7]	PG2d
pssA1M271	Macerated buds	Los Lagos	−40.378504, −73.042016	*P. syringae* pv. *syringae*	GCF_022513805.1	[7]	PG2d
pssA1M273	Cankerous wood	Los Ríos	−39.653131, −72.966329	*P. syringae* pv. *syringae*	GCF_022513845.1	[7]	PG2d
pssA1M274	Cankerous wood	Los Lagos	−40.704688, −73.165155	*P. syringae* pv. *syringae*	GCF_022513865.1	[7]	PG2d
pssA1M275	Cankerous wood	Los Lagos	−40.751156, −73.051729	*P. syringae* pv. *syringae*	GCF_022513885.1	[7]	PG2d
pssA1M276	Cankerous wood	Los Lagos	−40.423977, −73.185541	*P. syringae* pv. *syringae*	GCF_022513905.1	[7]	PG2d
pss11116_B1	Unknown	O’Higgins	−34.586163, −71.013075	*P. syringae* pv. *syringae*	GCF_029383325.1	-	PG2d
pss9298.1	Unknown	O’Higgins	−34.586322, −71.016573	*P. syringae* pv. *syringae*	GCF_029383285.1	-	PG2d
pssHO7	Unknown	Maule	−35.497218, −71.649631	*P. syringae* pv. *syringae*	GCF_029383205.1	-	PG2d
pssS2_2	Unknown	Maule	−35.500447, −71.651811	*P. syringae* pv. *syringae*	GCF_029383215.1	-	PG2d
pssA1M264	Buds	Ñuble	−36.685648, −71.962978	*P. syringae* pv. *syringae*	-	This Study	PG2d
pssA1M284	Wood and buds	Los Lagos	−40.847713, −73.461868	*P. syringae* pv. *syringae*	-	This Study	PG2d
pssA1M290	Wood	Los Lagos	−40.651371, −72702953	*P. syringae* pv. *syringae*	-	This Study	PG2d
pssA1M291	Wood	Maule	−36.230804, −71.736220	*P. syringae* pv. *syringae*	-	This Study	PG2d
pssA1M307	Wood	Ñuble	−36.685648, −71.962978	*P. syringae* pv. *syringae*	-	This Study	PG2d
psA1M325	Wood	Araucanía	−37.728270, −72526409	*Pseudomonas syringae*	-	This Study	PG2b

**Table 2:** Summary of *Pseudomonas syringae* PG2d clusters defined by PopPUNK analysis. Each cluster includes the total number of strains, number of Chilean isolates, host distribution, number of Hop effectors (HOPs), major Exchangeable Effector Locus (EEL) effector, type III secretion system (T3SS) genotype, type VI secretion system (T6SS) subtype, frequency of the combined *cusCBA/copABCD* copper resistance operon, and number of plasmids detected.

	Total N° Strains	Chilean N° Strains	Host	N° HOPs	HOP EEL	T3SS T-PAI	T6SS	% cusCBA/copABCD Detected	N° Plasmids Detected
Cluster 1	37	14	*Prunus avium*: 33 (89.2%) *Prunus domestica*: 1 (2.7%) *Prunus* sp.: 1 (2.7%) *Prunus. dulcis*: 1 (2.7%) *Prunus persica*: 1 (2.7%)	17–20	HopA	T-PAI-1	i1, i1	42.86	5
Cluster 2	13	0	*Pr. avium*: 11 (91.7%) *Pr. domestica*: 1 (8.3%)	18–20	HopA	T-PAI-3	i1, i4b	100	0
Cluster 3	9	8	*Pr. avium*: 9 (100%)	18–19	HopA	T-PAI-3	i1, i4b	0	0
Cluster 4	6	0	*Phaseolus vulgaris*: 4 (57.1%) *Abelmoschus esculentus*: 1 (14.3%) Stream water: 1 (14.3%) *Ph. vulgaris* (Lesotho): 1 (14.3%)	20–23	HoB-like HopZ3	T-PAI-1 T-PAI-3	i1, i4b	0	0
Cluster 5	6	0	*Pr. avium*: 6 (100%)	18–19	HopA	T-PAI-3	i1, i4b	100	0
Cluster 6	5	4	*Pr. avium*: 4 (80.0%) *Prunus cerasus*: 1 (20.0%)	18–19	HoB-like HopZ3	T-PAI-1 T-PAI-3	i1, i4b	0	0
Cluster 7	5	0	*Pr. avium*: 4 (80.0%) *Pr. cerasus*: 1 (20.0%)	20	HopA	T-PAI-3	i1, i4b	0	0
Cluster 8	4	0	*Ph. vulgaris*: 4 (100%)	21–24	HopA	T-PAI-3	i1, i4b	0	0
					HoB-like				
Cluster 9	4	0	*Ph. vulgaris*: 4 (100%)	20–21	HopZ3	T-PAI-1	i1	0	0
Cluster 10	3	0	*Pyrus communis*: 3 (100%)	16–17	HopA	T-PAI-1	i1, i4b	0	0
Cluster 11	3	3	*Pr. avium*: 3 (100%)	18	HoB-like	T-PAI-1	i1, i4b	0	3
Cluster 12	3	2	*Pr. avium*: 2 (66.7%) Lake water: 1 (33.3%)	19	HoB-like HopA	T-PAI-1	i1, i4b	0	0
Cluster 13	3	3	*Pr. avium*: 3 (100%)	16–17	HopA	T-PAI-2	i1, i4b	0	0
Cluster 14	3	1	*Pr. avium*: 2 (66.7%) *Ph. vulgaris*: 1 (33.3%)	19–22	HopA HoB-like HopZ3	T-PAI-1	i1, i4b	0	0
Cluster 15	3	1	*P. avium*: 2 (66.7%) *Pyr. communis*: 1 (33.3%)	16–19	HoB-like	T-PAI-1	i1, i4b	0	0
Cluster 16	2	2	*Pr. avium*: 2 (100%)	19	HopZ3	T-PAI-1	i1, i4b	0	2
Cluster 17	2	0	*Pr. avium*: 2 (100%)	20	HopA	T-PAI-1	i1,i1	100	1
Cluster 18	2	0	*Pr. avium*: 2 (100%)	20	HopA	T-PAI-3	i1, i4b	0	0

## Data Availability

The datasets generated during and/or analyzed during the current study are available in the NCBI repository as bioproject PRJNA750090. All *Pss* strains are deposited at the Chilean Collection of Microbial Resources and are available upon request at https://www.cchrgm.cl.

## Supplementary Materials

The following supporting information can be downloaded at https://www.mdpi.com/article/10.3390/plants15040552/s1, Table S1. Reference genomes used for comparative genomic analyses of *Pseudomonas syringae* and related species; Table S2. Distribution of type III secretion system (T3SS) and Exchangeable Effector Locus (EEL) genotypes among *Pseudomonas syringae* pv. *syringae* and related isolates within the PG2 phylogenetic group; Table S3. Comparative distribution of virulence, toxin, and copper resistance genes among *Pseudomonas syringae* phylogenetic groups PG2a–PG2d.

## Abbreviations

The following abbreviations are used in this manuscript:

ANI	Average Nucleotide Identity
CEL	Conserved Effector Locus
COG	Cluster of Orthologous Groups
EEL	Exchangeable Effector Locus
HMM	Hidden Markov Model
*hrp/hrc*	Hypersensitive Response and Pathogenicity/HRP Conserved genes
IAA	Indole-3-acetic Acid
INA	Ice Nucleation Activity
I1/I4a	Subtypes of Type VI Secretion Systems
ORF	Open Reading Frame
PAI	Pathogenicity Island
PCA	Principal Component Analysis
PG	Phylogenetic Group
PG2d	Phylogenetic Group 2d (subgroup within *Pseudomonas syringae*)
*Pss*	*Pseudomonas syringae* pv. *syringae*
T1SS	Type I Secretion System
T2SS	Type II Secretion System
T3SS	Type III Secretion System
T5SS	Type V Secretion System
T6SS	Type VI Secretion System
T3SE	Type III Secreted Effector
T-PAI	Tripartite Pathogenicity Island
*Pyr.*	*Pyrus*
*Pr.*	*Prunus*
*Ph.*	*Phaseolus*
